# Autophagy: A Potential Therapeutic Target for Reversing Sepsis-Induced Immunosuppression

**DOI:** 10.3389/fimmu.2017.01832

**Published:** 2017-12-18

**Authors:** Chao Ren, Hui Zhang, Tian-tian Wu, Yong-ming Yao

**Affiliations:** ^1^Trauma Research Center, First Hospital Affiliated to the Chinese PLA General Hospital, Beijing, China; ^2^School of Medicine, Nankai University, Tianjin, China; ^3^State Key Laboratory of Kidney Disease, The Chinese PLA General Hospital, Beijing, China

**Keywords:** sepsis, immunosuppression, autophagy, apoptosis, treatment

## Abstract

Sepsis remains the leading cause of mortality in intensive care units and an intractable condition due to uncontrolled inflammation together with immune suppression. Dysfunction of immune cells is considered as a major cause for poor outcome of septic patients but with little specific treatments. Currently, autophagy that is recognized as an important self-protective mechanism for cellular survival exhibits great potential for maintaining immune homeostasis and alleviating multiple organ failure, which further improves survival of septic animals. The protective effect of autophagy on immune cells covers both innate and adaptive immune responses and refers to various cellular receptors and intracellular signaling. Multiple drugs and measures are reportedly beneficial for septic challenge by inducing autophagy process. Therefore, autophagy might be an effective target for reversing immunosuppression compromised by sepsis.

## Introduction

**Figure 1:**
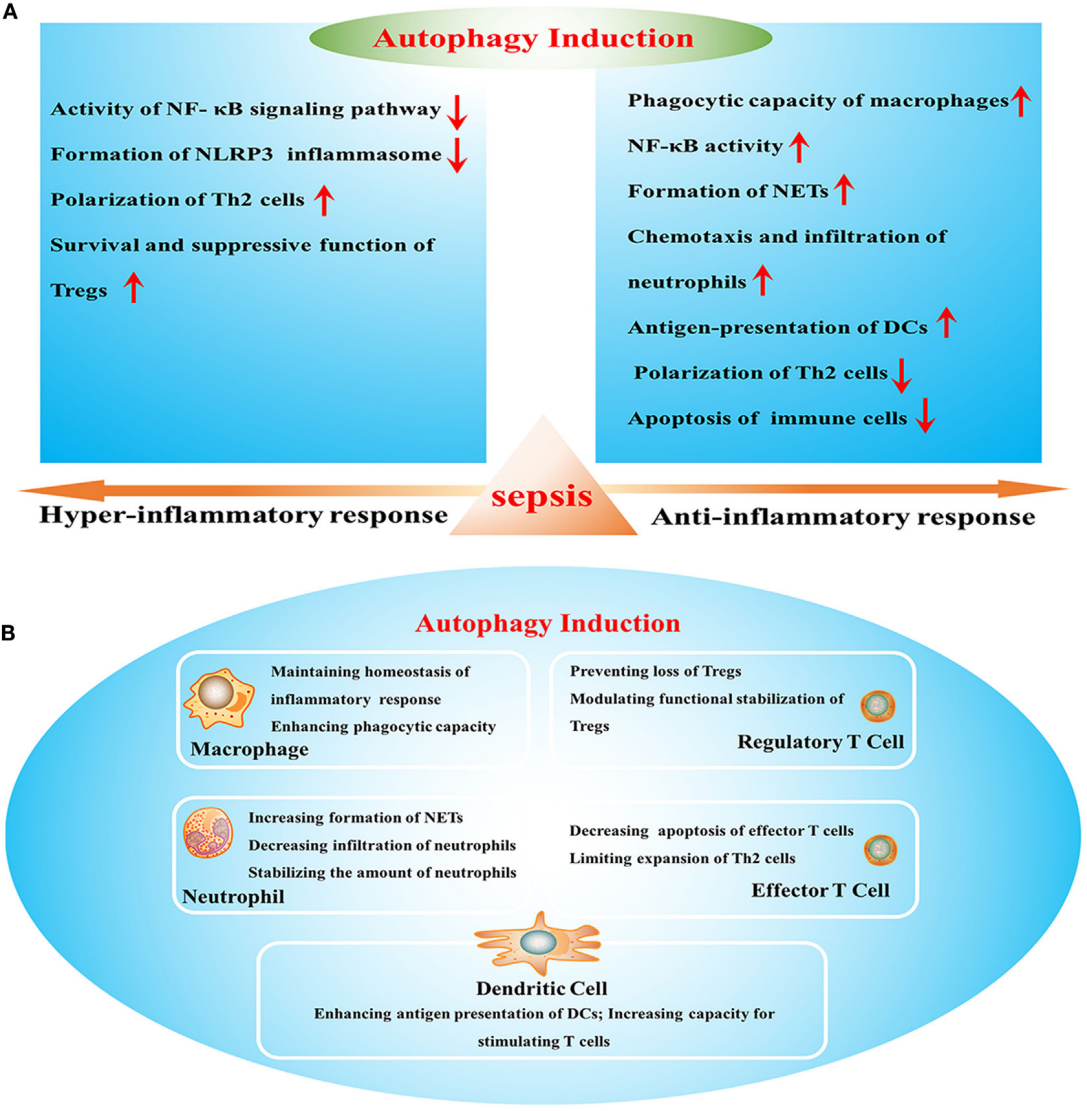
The effects of autophagy induction on sepsis-induced immune dysfunction. **(A)** Effects of autophagy in various stages of sepsis. **(B)** The role of autophagy induction in the function of multiple immune cells. NF-κB, nuclear factor kappa-light-chain-enhancer of activated B cells; NLRP3, Nod-like receptor family pyrin domain-containing 3; Tregs, regulatory T cells; NETs, neutrophil extracellular traps; DCs, dendritic cells.

Sepsis arises when hosts response to infection injuries to their own tissue and organ, which is manifested by uncontrolled inflammatory response and multiple organ failure. It acts as one of the leading causes of mortality in intensive care units (ICUs) and brings about millions of deaths a year (1). Current definitions for sepsis focus on organ dysfunction compromised by dysregulated response to infection and long-term outcomes of septic patients that survive from serious stage owing to sophisticated care in ICU (2). It has been reported that septic survivors showed higher readmission rate and more severe situations than those without septic experience (3). Uncontrolled inflammatory response and refractory immune suppression are considered as major causes for poor outcome of septic patients, which exist concomitantly but dominate in different stages (4). Sepsis-induced immunosuppression that covers both innate and adaptive immune systems acts as a predominant cause of late mortality due to recurrent infections (5). It presents with low levels of both pro- and anti-inflammatory mediators and massive depletion of immune cells, including monocytes, dendritic cells (DCs), B cells, and CD4^+^ and CD8^+^ T cells, in turn contributing to multiple organ failure as a result of high bacterial load and opportunistic infection (6, 7). Therefore, it will be of great significance to clarify specific mechanisms underlying inflammatory reprogramming and immunologic paralysis under septic exposure. Recently, upregulation of autophagic activity reportedly can influence the inflammatory response and the survival as well as function of immune cells (Figure 1) and show great benefits by ameliorating organ dysfunction and improving outcomes (8). Suppression or deficient of autophagy leads to dysfunction and depletion of immune cells, followed by disturbed immune immunity and increased mortality in septic condition (9, 10), indicating that autophagy might be an effective therapeutic target for sepsis.

Autophagy is an important self-protective mechanism for cellular survival by controlling degradation of proteins and organelles, which involves the formation of double-membrane autophagosome and the proteolytic degradation after delivered to lysosome. Studies have shown that autophagy is mobilized early in sepsis and seen in various organs, manifested by increased accumulation of autophagic vacuoles and enhanced expression of autophagy-associated proteins (11). For example, microtubule-associated protein light chain 3 (LC3), one of key molecules for autophagy, showed enhanced expression at 6 h after cecal ligation and puncture (CLP) and released a cascade of benefits by eliminating invaded pathogens, avoiding overproduction of stress proteins and modulating the function of multiple organelles (12). SQSTM1/p62, another specific marker for dynamic autophagic process that is also termed autophagic flux, revealed rapid alteration after sepsis initiation and further contributed to functional stability of various cells (11). However, a host in recent studies found disturbed autophagic process in later stage of sepsis, which was considered as a major cause for sepsis-induced immune suppression (10). In this review, we will provide a detailed overview of the effects of autophagy on immune response and further therapeutic significance in sepsis.

## The Protective Role of Autophagy in Sepsis

Autophagy initiates early after the onset of sepsis and is induced by some kinds of bacteria, bacterial toxins such as lipopolysaccharide (LPS), and pro-inflammatory cytokines (13–15). The intracellular signaling that is responsible for autophagy induction mainly includes 5′ adenosine monophosphate-activated protein kinase and c-Jun *N*-terminal kinase/p38 mitogen-activated protein kinase (MAPK) pathways under activation of Toll-like receptor (TLR) 4 and TLR9, respectively (16, 17). It has been demonstrated that autophagy confers protective effects on multiple organs and systems in septic challenge, involving heart, liver, lungs, kidneys, brain, and coagulation system (11, 18–22). Liver dysfunction, for instance, presents with extensive apoptosis of hepatocytes and elevated serum levels of aminotransferases during sepsis, which can be reversed by autophagy activation (23). Further evidence has shown that maintaining mitochondrial homeostasis contributes to one of the major mechanisms of autophagy on ameliorating hepatocyte injury (24). Such mechanism equally applies to the protective effects of autophagy on lung epithelial cells and cardiomyocytes, which relate to the quality control of mitochondria and endoplasmic reticulum (25, 26), indicating that the homeostasis of multiple organelles constituted by autophagy induction may become effective targets for improving outcomes in the setting of sepsis. Actually, selective degradation of organelles has been recognized with distinct terminology, such as nucleophagy, reticuluophagy, lysophagy, mitophagy, and pexophagy, and further noted for the development of various diseases after the discovery of distinct molecules.

The formation of autophagic vacuoles and the expression of autophagy-associated proteins vary with different tissues or organs and different stages of sepsis. LC3, for example, was found with enhanced expression at 8 h after CLP operation but declined to baseline value at 12 h in intestinal epithelial cells (12). It presented with two expression peaks at 6 and 36 h in renal tissues under sepsis exposure (27). This tendency was reportedly similar to the expression of Beclin-1, another key modulator of autophagic process (12). These discrepant results might attribute to the different states of multiple organs, as restoration of autophagy mainly resulted in protective effect by maintaining functional homeostasis. As with autophagic vacuoles, double-membrane formatted structure that is direct evidence of autophagic action, its amount also changes over time during septic course. It increased in hyperdynamic phase, from 4 to 9 h post-CLP surgery, but showed significant reduction from 18 to 24 h, implicated with hypodynamic phase (9). Atg7, an essential gene for autophagosome formation and reportedly beneficial for immune homeostasis, also exhibited the similar tendency, as its expression was enhanced at 8 h but significantly suppressed at 24 h post-CLP surgery (9, 12). Recently, autophagic flux is documented to represent dynamic function of autophagy that can be evaluated by expressions of Beclin-1, LC3, and p62 and plays a protective role in immune cells with completed autophagic process (28).

## Autophagy and Immune Response

**Figure 2:**
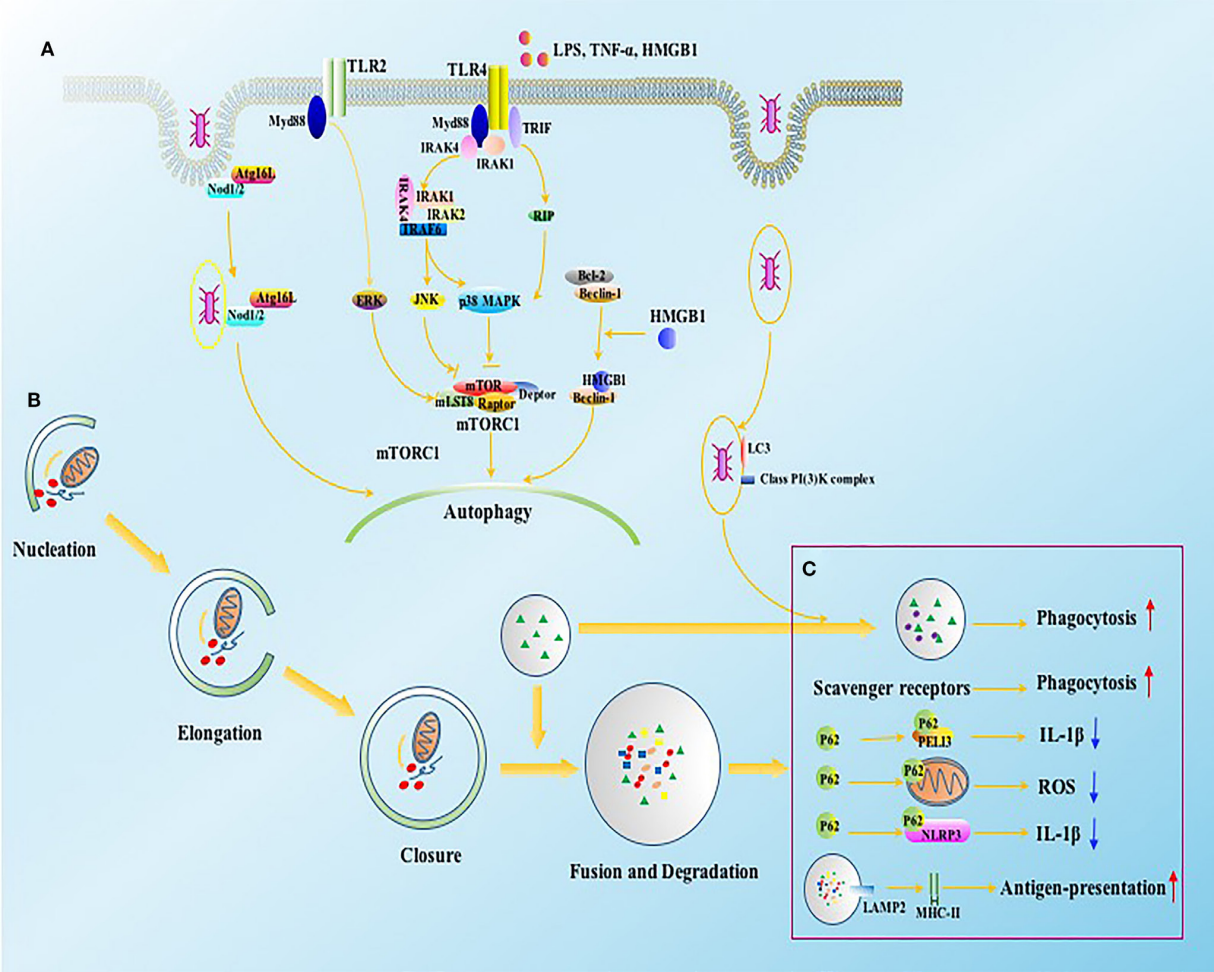
The intracellular signals of autophagy induction in immune cells. **(A)** Autophagy can be induced by bacteria, bacterial toxins such as LPS, and pro-inflammatory cytokines through TLR2 and TLR4. The activation of TLR4 and TLR2 initiate JNK, p38 MAPK, and ERK signaling in Myd88 and TRIF dependent ways, which further induce autophagy by inhibiting mTORC1 complex. NOD1 and NOD2 receptors, vital intracellular pattern recognition receptors, promote the formation of autophagosomes by enhancing the combination between ATG16L1 and invaded bacteria. HMGB1 induces autophagy through releasing Beclin-1 from Bcl2 after binding with Beclin-1. **(B)** Four major steps are required for autophagic process, including nucleation, elongation, closure, and fusion with lysosome for degradation, which are tightly regulated by autophagy associated proteins. **(C)** The function of autophagy associated proteins on immune system involves both canonical and non-canonical dependent ways. ATG16L1, autophagy-related 16-like 1 gene; Bcl2, B-cell lymphoma 2; ERK, extracellular signal-regulated kinase; HMGB1, high-mobility group box-1 protein; IL, interleukin; IRAK, interleukin-1 receptor-associated kinase; JNK, c-Jun N-terminal kinase; LAMP, lysosomal-associated membrane protein; LPS, lipopolysaccharide; MHC, major histocompatibility complex; mLST8, mammalian lethal with SEC13 protein; mTOR, mammalian target of rapamycin; mTORC1, mTOR complex 1; Myd88, myeloid differentiation factor 88; NOD, nucleotide-binding oligomerization domain-containing protein; PELI3, pellino E3 ubiquitin protein ligase family member 3; p38 MAPK, p38 mitogen-activated protein kinase; RIP, receptor interacting protein; ROS, reactive oxygen species; TNF, tumor necrosis factor; TRAF, TNF receptor-associated factor; TRIF, TIR-domain-containing adapter-inducing interferon-β.

Autophagy has been reported to play a crucial role in modulating immune response since its pathogen-killing potential was noticed by Nakagawa et al. in 2004 (13). Both autophagic process and independent autophagy-associated proteins are involved in eliminating invaded pathogens and maintaining immune homeostasis through altering phagocytosis, antigen presentation, and differentiation of immune cells (Figure 2) (29–32). Autophagy deficient mice, however, showed disturbed eradication of invaded pathogens and damaged organelles and were more sensitive to bacterial infection (29). In addition, various autophagy-related proteins function differently in the course of phagocytosis. LC3, for example, can be recruited to phagosomes and further facilitates the clearance of pathogens and dead cells in a canonical autophagy-independent way, suggesting that autophagy-associated proteins might be effective therapeutic targets for immune response other than mediating autophagic process (33). The same goes for the antigen-presenting function, as autophagy-mediated antigen presentation is important for priming activation of CD4^+^ T cells during infection (34). Mice with Atg5 deficient in DCs exhibited impaired activities of CD4^+^ T cells as a result of reduced interleukin (IL)-2 and interferon (IFN)-γ production, but without affecting costimulatory molecule expressions on DCs, which were different from Beclin-1 and vacuolar protein sorting 34 (Vps34). The DCs exhibited long-standing dysfunction and massive loss in animals with Beclin-1 or Vps34 deficient (34–36). However, Bhattacharya and colleagues found that the loss of Atg7 gene displayed protective effects against autoimmune encephalomyelitis in mice (37). These conflicting results may also arise from diverse features of autophagy-related proteins in various disease states. However, the specific mechanism with regard to autophagy activation in modulating immune function upon sepsis exposure remains to be ascertained. In the remaining part, we will discuss the pleiotropic effects of autophagy on the function of immune cells in septic states.

## Neutrophils

Neutrophil belongs to the body’s first defensive system and is vital for pathogen clearance through effective phagocytosis, degranulation, and formation of neutrophil extracellular traps (NETs) (38). Neutrophil recruitment, another significant feature of immune response, is considered as a prerequisite for its infection control, which requires multiple chemokines and chemotaxis receptors (39). The migration of neutrophils can be initiated by various factors, including low concentrations of LPS and non-severe CLP operation, which are reportedly TLRs dependent (40). Similarly, lipoteichoic acid, the major toxin from Gram-positive bacteria, also provokes the activation of neutrophils, accompanied by massive cytokine release (41). However, disturbed or inappropriate migration of neutrophils appears to be a great threat of lethal sepsis, manifested by uncontrolled bacterial load and multiple organ dysfunction (42). The failure of neutrophil migration is commonly observed in overdose LPS stimulation and septic challenge, which may result in poor outcome (40, 42). Simultaneously, expressions of chemotactic mediators were markedly enhanced in infection sites, indicating that the depressed neutrophil migration might not result from the loss of chemokines (43). In fact, overproduction of chemokines and inflammatory cytokines has been reportedly detrimental to neutrophil migration, tumor necrosis factor (TNF)-α, for example, and it reduced neutrophil chemotaxis by downregulating expressions of CXC chemokine receptor (CXCR) 1 and CXCR2 (44). While mice with TNF receptor deficient revealed high CXCR2 expression and enhanced neutrophil chemotaxis under sepsis exposure (45). Inappropriate infiltration of neutrophils resulted in the remote organ dysfunction in sepsis, which was further identified as C-C chemokine receptor type 2 (CCR2) dependent (46). Targeting CCR2 with specific antagonist or gene blockade showed protective effects on lungs, heart, and kidneys and improved survival rate of septic animals by lowering infiltration of neutrophils (46).

Autophagic machinery has been documented to enhance the bactericidal activity of neutrophils from immunocompromised patients, while it can be reversed by autophagy inhibition (47, 48). Both intracellular and extracellular mechanisms are involved in this effect, the former is characterized by killing invaded intracellular bacteria, while ${\text{O}}_{2}^{-}$ releases and NETs formation are major factors for the later, which show great benefits for septic cases (48, 49). Further studies suggested that phosphatase and tensin homolog on chromosome ten, a dual phosphatase that activated autophagic machinery by antagonizing Akt/mammalian target of rapamycin (mTOR) pathway, increased NETs formation upon activation, implying that autophagy might be a potential target for improving bacterial killing potential of neutrophils (50). Indeed, mTOR has been documented as an essential intracellular pathway for NETs formation by regulating autophagy activation (51). Autophagy can also regulate the chemotaxis and infiltration of neutrophils, which are major causes for multiple organ dysfunction after disrupted by lethal sepsis, as mentioned above (40). Induction of autophagic process decreased neutrophil infiltration and further alleviated lung injury and improved survival rate following sepsis, while Atg4b-deficient animals presented with exaggerated lung injury together with high mortality rate (52, 53). As excessive production of inflammatory mediators is responsible for disturbed neutrophil migration, stimulation of autophagy might also benefit its migration by downregulating the expression of inflammatory cytokines. Moreover, autophagy can stabilize the amount of neutrophils by regulating the apoptosis compromised by inflammatory stimuli (54).

## Monocytes and Macrophages

Monocytes and macrophages are major representatives of innate immune system, which function as the first line of immune defense system. They both initiate early and show host defense by eradicating invaded pathogens or damaged tissues and releasing pro-inflammatory cytokines (55). However, excessive production of pro-inflammatory mediators has been identified as one of the major causes for high mortality rate in early phase of sepsis (1). Current opinions on the progression of sepsis focus on distinguishing the dominance between pro- and anti-inflammatory responses in various stages, which calls for different treatments (56). Further investigations have reported that late deaths in sepsis mainly resulted from ongoing infection, involving both unresolved and opportunistic infections (57). The dysfunction of monocytes and macrophages, as illustrated by Monneret et al. (58), was indeed the major cause for increased secondary infection after sepsis induction, due to disturbed capacity in eliminating invaded pathogens and releasing pro-inflammatory cytokines (58, 59). In addition to decreased release of pro-inflammatory mediators, such as TNF-α, IL-6, and IL-1β, the anti-inflammatory cytokines, IL-10 and transforming growth factor (TGF)-β, for example, aggravated the suppressed function of macrophages and monocytes with elevated production (60). Moreover, human histocompatibility leukocyte antigen (HLA)-DR, an important molecule for antigen presentation of monocytes, is another key marker for immune suppression when it shows significantly declined expression, which is responsible for impairing proliferation of T cells and augmenting helper T cell (Th) 2 polarization (61, 62). Therefore, modulating homeostasis of pro- and anti-inflammatory responses and functional stabilities of monocytes as well as macrophages can be of great benefits for improving outcome associated with septic complications.

The protective mechanism of autophagy is partly due to its regulatory potential of inflammation, as presented in multiple kinds of inflammatory states (63). In the early sepsis, plenty of inflammatory mediators are urged to cope with high bacterial load and toxic insults, which call for rapid mobilization and activation of macrophages and monocytes. Concurrently, autophagic process of these cells initiates to maintain homeostasis of inflammatory response and cell survival, which changes over different stages (64). In a hyper-inflammatory situation, autophagy was needed to avoid excessive inflammatory response by presenting as an anti-inflammatory mechanism, and macrophages with suppressed autophagy exhibited excessive production of pro-inflammatory cytokines (32, 65). It has been demonstrated that the anti-inflammatory effects of autophagy mainly rooted in interfering the nuclear factor (NF)-κB signaling pathway and the formation of Nod-like receptor family pyrin domain-containing 3 inflammasome that were both responsible for induction of various inflammatory mediators (64, 66). Such kind of effect was identically seen in non-selective and selective autophagy of macrophages: macro-autophagy and mitophagy, respectively (66). When developing the immunosuppressive state, autophagy presented with great potential of immunomodulation to restore immune function. Induction of autophagy has been reported to decrease blood bacterial load through enhancing phagocytic capacity of macrophages, followed by attenuating organ injury and improving survival rate (67). Interestingly, chloroquine, an inhibitor of autophagic process, was noted to decrease serum high-mobility group box-1 protein levels by disturbing NF-κB activity and further rescued mice from lethal sepsis, indicating a positive effect of autophagy on NF-κB signaling (68). The activity of NF-κB could be affected by autophagy, which involved both upregulation and downregulation, as Criollo et al. (69) demonstrated that autophagy was required for the TNF-α-induced activation of NF-κB. The activation of NF-κB revealed a negative effect on autophagic process by enhancing the activity of mTOR (70). Actually, the interplay between NF-κB signaling and autophagy is a quite complicated network, which varies with different cells and disease types (65, 69, 71). In the setting of sepsis, autophagy is currently considered as an effective therapeutic target for maintaining homeostasis of inflammatory response and restoring functions of macrophages as well as monocytes (19, 64, 67). Thus, further studies are expected to clarify precise mechanisms underlying the potential role of autophagy in the development of sepsis and to explore effective treatments by activation of autophagy.

## Dendritic Cells

Dendritic cells are known for great competence in antigen presenting and bridge innate and adaptive immune systems (72). In response to infection or damage stimuli, immature DCs that are characterized with phagocytic properties transform into mature states with enhanced antigen-presenting abilities to initiate the activation of T cells (73). The process of DC transformation is vital for immune response, which involves decreased phagocytic ability but elevated expressions of surface molecules, mainly including CD40, CD80, CD86, and major histocompatibility complex (MHC) II, which are necessary for the activation of T cells (73). In the setting of sepsis, however, DCs became abnormal in both numbers and function, manifested by massive loss and disturbed capacity for T cell stimulation, which was responsible for sepsis-induced immunoparalysis (74). The number of DCs reduced dramatically in both spleen and mesenteric nodes at 12 h after CLP operation (75, 76). Similarly, blood DCs of severe septic patients showed significantly depletion compared to the healthy controls (77). Apoptosis, a major cause for DC depletion, was significantly enhanced in lethal sepsis, which was further identified as TLR2 and TLR4 dependent, since it was prevented in TLR2^−/−^ and TLR4^−/−^ mice (75, 78). The dysfunction of DCs in septic condition is composed primarily of two parts: suppressed expressions of surface molecules and declined production of cytokines, both followed with suppressed activation of T cells (74). In line with the concept, expressions of CD80, CD86, and MHC II molecules on splenic DCs were dramatically reduced at 48 h following LPS challenge, accompanied by decreased formation of IL-12, IL-6, and TNF-α and further disturbed antigen presentation (79). In the clinical practice, HLA-DR and CD86 expressions on human DCs have been reported to obviously downregulate in septic patients compared with the controls (80). IL-12, secreted by DCs, is another major factor for T cell activation and contributes to depressed proliferation and altered differentiation of T cells when presents with reduced production in lethal sepsis (81). Other factors that contribute to the negative connection between DCs and activation of T lymphocytes involve enhanced expression of negative costimulatory molecules. For instance, expressions of programmed cell death 1 ligand 1 (PD-L1) and PD-1 were reportedly upregulated in mice with sepsis/multiple organ dysfunction syndrome (MODS) and were closely related to tolerant DCs and development of MODS, as blockade PD-L1 and PD-1 signaling significantly reversed above disadvantages (82).

The autophagy does function as an immunomodulatory mechanism for DCs, as it exhibits disturbed antigen processing and presentation and fails to prime T cells with deficient of autophagy-associated genes, such as Atg16, Atg7, and Beclin-1 (37, 83, 84). Autophagy activation has been proved to enhance antigen presentation of DCs by increasing expression of MHC II molecules, suggesting that autophagy induction possesses a critical role in maintaining DC function (85). In addition, the effects of autophagy-associated genes varied with different types, Beclin-1, for example, Beclin-1^+/‒^ DCs revealed suppressed expression of MHC II molecules and pro-inflammatory cytokines and further induced Th2 polarization in comparison with wild controls, indicating a protective role of Beclin-1 in maintaining homeostasis of immune response (84). Likewise, DCs with autophagy-related 16-like 1 (Atg16L1) gene deficiency showed excessive production of reactive oxygen species and abnormal antigen processing under colitis exposure (83). Yet interestingly, Atg16L1 was found to avoid lethal T cell alloreactivity by inhibiting the immune response of DCs in graft-versus-host disease, suggesting that the protective role of autophagy in DCs varied with disease types (86). Nevertheless, limited studies have focused on investigating the impact of autophagy on DC dysfunction secondary to septic response, which might be advantageous to explore potential targets for the treatment of sepsis.

## T Lymphocytes

T cells are recognized as key elements of adaptive immune system and mainly divided into three subtypes according to produced cytokines. Th cell, the most abundant one, is reported as the major victim under septic exposure, which suffered from massive depletion and obvious dysfunction (56). For example, T cell exhaustion, resulted from intensified apoptosis, was inevitably accompanied by decreased survival rate in septic mice models and clinical patients (87). Likely, clinical studies indicated that patients dying of sepsis experienced striking loss of both CD4^+^ and CD8^+^ T cells, which were primary induced by apoptosis (88). Antiapoptotic measures, as documented by Wesche-Soldato et al. (89), were proven to be highly effective in mitigating death of lymphocytes and improving survivals of experimental models. Multiple factors reportedly contributed to the intensified apoptosis of T cells in septic condition (82, 90). PD-L1 significantly accelerated T cell apoptosis after boned with PD-1, while administration of PD-L1 antibody markedly attenuated T lymphocyte apoptosis and improved survivals of septic mice (82). Recently, polarization of Th cells, another important mechanism for alteration of immune state, also deserves attention. Th1, Th2, and Th17 cells are major representatives of Th cells and are distinguished by secretion of various cytokines, which are closely associated with different types of inflammatory response (91). Th1 cells, e.g., present with pro-inflammatory response by secreting counterpart mediators, including IL-1β, TNF-α, and IFN-γ. Th2 cells, on the contrary, are inclined to anti-inflammatory response with elevated production of anti-inflammatory cytokines, such as IL-4, IL-5, and IL-10. Actually, the ratio of IFN-γ/IL-4 has been identified as a good marker for immune state by reflecting the dominated differentiation between Th1 and Th2 cells in septic state (92). Multiple factors that are raised during septic course result in the discrepant differentiation of Th cells and of particular note is regulatory T cells (Tregs). Tregs are specified in immunosuppressive function and can inhibit activation and proliferation of T cells and induce Th2 polarization by direct contacting with the actions of forkhead/winged helix transcription factor p3 (Foxp3) as well as cytotoxic T lymphocyte-associated antigen-4 or secreting anti-inflammatory mediators, such as IL-10 and TGF-β (93). Proper downregulation of Treg numbers, as documented by Heuer and colleagues (94), improved proliferation of Th cells and enhanced Th1 differentiation. Nonetheless, depletion of Tregs was reported to serve no benefits to the survivals of septic mice, implying that maintaining activities of Tregs in a certain range appeared to be important for resolving sepsis (95). Taken together, precision modulation of the Th1/Th2 polarization and the function of Tregs might be of great significance on restoring immune dysfunction subsequent to sepsis.

There is a growing body of evidence suggesting that autophagy has shown protective effects on T cells, as T-cell-specific Atg7-knockout mice presented massive loss of T cells and increased mortality after exposed to sepsis (9). Moreover, loss of T cells mainly resulted from upregulated expression of Bcl-2-like 11 and PD-1 after inhibition of autophagic process, indicating that there was a close relationship between autophagy and apoptosis (10). Under the physiology condition, autophagy induction thrives on stress adaption by suppressing apoptosis and cell death, but it initiates an alternative cell death after failed to survive from damage stimuli (96). In this respect, autophagy should be tightly regulated to maintain cell survival. In addition, the effects of autophagy on preventing T cell apoptosis could be partly due to its alternative role on NF-κB activation, as enhanced NF-κB activation retarded the depletion of T cells and further decreased mortality in murine sepsis (21, 97). Suppression of autophagy also augmented the release of anti-inflammatory cytokines in T cells; for example, IL-10, further induced a tendentious differentiation to Th2 cells (10). Recently, autophagy induction was documented to limit Th2 cell expansion through altering metabolic profile, while enhanced Th2 response was noticed in Atg16l1 depletion mice (98). Furthermore, autophagy appeared to be responsible for survival and linage stability of Tregs, and as documented by Wei et al. (99), it revealed marked apoptosis and depressed expression of Foxp3 after Treg-specific gene knockdown of Atg7 or Atg5, which mechanistically referred to the excessive activation of T-cell receptor (TCR)-mTOR complex 1 (mTORC1) signaling under autophagic depletion. Interestingly, stimuli for mTORC1 activation that was inputted by TCR signaling has been identified as crucial regulators for the suppressive function of Tregs, since inhibition of mTORC1 resulted in a remarkable loss of Tregs (100). Therefore, either overactivation or disruption is detrimental to the suppressive activity of Tregs. Notch1 pathway is also required for Treg autophagy by coupling Beclin-1 and Atg14 with Notch intracellular domain and further regulates cell survival (101). However, little data are available concerning the potential role of autophagy in mediating immune response of Tregs secondary to septic challenge, and it is probably because of the limited Treg numbers and undetermined mechanisms for autophagic functions. Given the gradually clarified association between autophagy and function of Tregs, it is for certain that autophagy will be an effective target for modulating functional stabilization of Tregs.

## Clinical Perspectives and Conclusion

The clinical significance of autophagy has been recognized since Beclin-1 deficient was first identified as a cause for tumorigenesis by Qu et al. in 2003 (102), which further promoted an upsurge in research of autophagy. Rapamycin, the first drug discovered for autophagy induction, exerted a protective effect on multiple diseases, such as cancer, neurodegenerative disease, and infection. Up to now, little progress has been made in clinical use of autophagic regulators, while major achievements are stacked on cellular autophagy. Indeed, it is truly difficult to provide specific treatments on autophagy due to diverse effects of autophagy-associated proteins and undetermined role in various disease settings, especially for septic response. Recently, various kinds of drugs that are applied to reverse sepsis-induced immunoparalysis have been identified as autophagy-dependent (Table 1), which provide a bright prospect of autophagic modulation in clinical application (103, 104). Therefore, more studies are expected to explore specific mechanisms of autophagy under different pathological exposures.

**Table 1:** The effects of different drugs on sepsis by inducing autophagy.

Names	Effects	Reference
Globular adiponectin	Prevent IL-1β formation, inhibit caspase-1 activation, and reduce cell death	(16)

Sinomenine hydrochloride	Improve survival of CLP mice, attenuate multiple organ injury, and reduce the production of systemic inflammatory mediators	(8)

Carbon monoxide	Increase the survival of CLP mice, downregulate pro-inflammatory cytokines, diminish bacterial load, and enhance bacterial phagocytosis of macrophages	(67)

EGCG	Improve the survival rate of septic mice and inhibit HMGB1 expression and extracellular release	(103)

Hydroxytyrosol	Ameliorate pulmonary edema, reduce infiltration of inflammatory cells, and downregulate the expression of pro-inflammatory mediators	(104)

Ghrelin	Prevent intestinal mucosa injury and decrease MCP-1 levels in serum and intestinal tissues.	(12)

Genipin	Improve survival rate of CLP mice, decrease serum levels of aminotransferases, and reduce production of pro-inflammatory cytokines	(23)

*CLP, cecal ligation and puncture; HMGB1, high-mobility group box-1 protein; IL, interleukin*.

Autophagy is a typical self-protective mechanism not only for immune cell survival but also shows benefits for maintaining functional homeostasis of host immune system in septic settings. Reportedly, the kinetics of autophagy changed with time course and severity of sepsis and showed close relationship with the development of MODS and augmented mortality when endured with functional disruption (11). Induction of autophagy did alleviate sepsis-induced immunosuppression, which covered both innate and adaptive immune systems (9). It should be noted that the effects of autophagy might vary with different types of immune cells and refer to multiple cellular receptors as well as intracellular signaling pathways. Therefore, it will be of great significance to explore potential therapeutic targets that are concerned with autophagy for reversing immunosuppression compromised by sepsis.

## Author Contributions

CR and HZ conducted the literature review and drafted the manuscript. T-tW provided critical writing in the revised manuscript. Y-mY designed and instructed the writing of the manuscript.

## Conflict of Interest Statement

The authors declare that the research was conducted in the absence of any commercial or financial relationships that could be construed as a potential conflict of interest.
